# Comparative Genomic Insights into MatE Transporter Diversity and Habitat Adaptation of Archaea

**DOI:** 10.3390/microorganisms14030531

**Published:** 2026-02-25

**Authors:** Huan Leng, Leizhou Guo, Yi Chen, Liping Bai, Guihong Cha, Frank Delvigne

**Affiliations:** 1 Key Laboratory of Development and Application of Rural Renewable Energy, Biogas Institute of Ministry of Agriculture and Rural Affairs, Chengdu 610041, China; lenghuan@caas.cn (H.L.); 19934274758@163.com (Y.C.); 2 Terra Research and Teaching Centre, Microbial Processes and Interactions (MiPI), Gembloux Agro-Bio Tech, University of Liège, 5030 Gembloux, Belgium; f.delvigne@uliege.be; 3 Chengdu Institute of Food Inspection, Chengdu 611130, China; guoleizhou@caas.cn

**Keywords:** archaea, MatE transporters, habitat adaptation, metabolic type

## Abstract

Archaea comprise deeply rooted and phylogenetically diverse lineages that inhabit a wide range of environments and play essential roles in global biogeochemical cycles. However, the diversity of MATE (Multidrug and Toxic Compound Extrusion) family transporters in archaea, which are presumably involved in habitat adaptation, remains poorly understood. Here, we systematically analyzed archaeal MatE transporters using large-scale phylogenetic and comparative genomic analyses, combined with structure-based clustering and molecular docking. Our results show that MatE transporters are significantly enriched in archaea from host-associated and hypersaline environments compared with those from other habitats. Specific MatE transporters are strongly associated with particular habitats, and their copy numbers are positively correlated with genome size. Moreover, MatE transporters in archaea exhibit structural diversity and can be classified into four structural classes, among which Class I is predominant in both abundance and phylogenetic distribution compared with Classes II, III, and IV. Overall, these findings indicate that the successful adaptation of archaea to specific habitats is related to the acquisition and maintenance of MatE transporters, which may be critical for their survival in these environments.

## 1. Introduction

Archaea represent one of the most ancient and deeply rooted lineages of life and exhibit remarkable diversity in metabolic strategies, cellular physiology, and ecological distribution [1,2]. Early studies primarily associated archaea with extreme environments, such as highly acidic, saline, or high-temperature habitats [3], which strongly shaped the traditional view of archaea as extremophiles. However, with the rapid development of culture-independent approaches and genome-resolved metagenomics [4,5], it has become increasingly evident that archaea are not confined to extreme niches but are instead widely distributed across a broad range of natural and engineered environments. These include host-associated, hypersaline, freshwater, bioreactor, terrestrial, hydrothermal vent, marine, hot spring, cold spring, and oil field environments [6,7,8,9,10]. This extensive ecological distribution suggests that archaea have evolved diverse and flexible molecular mechanisms to cope with complex and fluctuating environmental conditions.

Among the cellular systems that underpin archaeal environmental adaptability, membrane transport systems play a particularly critical role. Membrane transporters are essential for maintaining cellular homeostasis, facilitating nutrient uptake, eliminating toxic compounds, and regulating osmotic balance [11,12], thereby enabling microorganisms to survive in environments with highly variable chemical and physical properties. The MATE (Multidrug and Toxic Compound Extrusion) family comprises a group of integral membrane proteins that are widely distributed across prokaryotes and eukaryotes [13,14]. Members of this family have been implicated in a broad range of biological functions, including the extrusion of xenobiotics and toxic compounds, accumulation of secondary metabolites, detoxification of herbicides, translocation of metal ions such as Fe^2+^, hormone signaling, aluminum (Al) transport, and participation in host–pathogen interactions [11,15,16,17,18,19]. Despite their functional importance in other domains of life, MatE transporters in archaea remain poorly characterized, and their diversity, evolutionary distribution, and potential roles in environmental adaptation have not yet been systematically investigated.

In our previous study, we demonstrated that the methanogenic archaeon *Methermicoccus shengliensis* employs MATE family transporters to mediate the uptake of methoxylated aromatic compounds from the extracellular environment, thereby establishing a direct link between MatE transporters and archaeal energy metabolism and methanogenesis [20]. This finding provides experimental evidence that MatE transporters can play functionally significant roles in archaeal physiology. However, whether such transporters are broadly distributed across archaeal lineages, how their occurrence varies among different ecological niches, and whether they exhibit diversity remain open questions.

In this study, we performed large-scale phylogenetic and comparative genomic analyses to investigate the distribution and diversity of MatE transporters across archaea. We show that MatE transporters are widely distributed among archaea and are significantly enriched in host-associated and hypersaline environments, occurring predominantly in heterotrophic, hydrogenotrophic, and methylotrophic archaea. Notably, the copy number of MatE transporters is positively correlated with genome size, suggesting that their expansion is jointly shaped by genome complexity and ecological selection. Furthermore, by integrating protein structural-based clustering and molecular docking analyses, we reveal clear structural diversity among archaeal MatE transporters, which can be classified into four distinct structural classes. These classes exhibit differences in key substrate-binding residues when interacting with identical substrates, providing structural insights into the potential functional diversification of MatE transporters. Collectively, our results highlight the contribution of MatE transporter diversity to archaeal environmental adaptation and metabolic flexibility.

## 2. Method Details

### 2.1. Genome Datasets

The sequences of archaea genomes were downloaded from NCBI (https://www.ncbi.nlm.nih.gov/genome/microbes/, accessed on 15 January 2024), JGI (https://img.jgi.doe.gov/, accessed on 15 January 2024), EBI-gut-rumen (https://www.ebi.ac.uk/metagenomics/genome-catalogues/human-gut-v2-0-2, accessed on 15 January 2024), and GTDB (https://gtdb.ecogenomic.org/, accessed on 15 January 2024) A total of 10,864 genomes were collected for downstream analyses. To obtain a more reliable genome dataset, we assessed the quality of each genome using CheckM2 (version 1.0.2) [21],which determines the estimated completeness of a genome and detects possible contamination based on lineage-specific sets of single-copy genes. We further compiled a high-quality dataset, including only genomes that were nearly complete (completeness ≥ 90%) and had low contamination (less than 5% contamination). All 10,864 genomes were subjected to quality control according to these criteria to reduce data redundancy and biased genome representation of Archaea.

### 2.2. Collection of Metadata

A variety of habitats were included in the analysis. Habitats of the 10,864 archaea strains were derived from their isolation source. The isolation source for each strain was determined manually by searching IMG metadata (https://img.jgi.doe.gov/, accessed on 18 May 2024), NCBI Biosample (https://www.ncbi.nlm.nih.gov/biosample/?term=, accessed on 18 May 2024), GDTB (https://gtdb.ecogenomic.org, accessed on 18 May 2024), ATCC (https://www.atcc.org, accessed on 18 May 2024), and the scientific literature. Based on their isolation sources, we categorized the genomes into host-associated, hypersaline environments, freshwater, bioreactor, terrestrial, hydrothermal vent, marine, hot spring, cold spring, and oil field sources; genomes whose source of isolation was not among these major habitats were labeled as belonging to other habitats; and genomes without isolation information were labeled as belonging to an unknown habitat.

### 2.3. Bioinformatics and Data Analysis Software

Phylogenetic trees were constructed using IQ-TREE2 (version 2.2.2.6) [22]. Homologous protein searches were performed using BLAST 2.15.0 against local genomes [23], and multiple sequence alignments (MSAs) were generated using MAFFT v7.526 with default parameters [24]. Protein topologies and domain architectures were predicted using SMART (https://smart.embl.de/, accessed on 23 June 2024) [25], CDvist (http://cdvist.utk.edu/, accessed on 23 June 2024) [26], and HHpred (https://toolkit.tuebingen.mpg.de/tools/hhpred, accessed on 23 June 2024) [27]. Phylogenetic trees were visualized and annotated using iTOL (http://itol.embl.de/, accessed on 16 December 2024).

### 2.4. Identification and Analysis of Protein

Genes encoding MatE transporters in the selected genomes were identified by BLASTP, with hits showing an E-value < 1 × 10^−5^ considered as candidates. The protein sequence of the MatE domains of transporters from *Methermicoccus shengliensis* DSM_18856 (BP07_RS03225) [20] were selected as query sequences to perform the BLASTP analysis. All candidate proteins were manually reexamined for domain organization using SMART, HHpred, and CDvist [25,26,27]. A complete list of identified MATE transporter proteins is provided in Table S2.

### 2.5. Phylogenetic Analysis

To construct a phylogenetic tree from the 10,864 archaeal genomes in our lab dataset (https://doi.org/10.5281/zenodo.18079802, accessed on 29 December 2025 and Table S1), multiple sequence alignments were generated by concatenating 122 phylogenetically informative protein- or protein domain-encoding sequences from the Pfam v27 [28] and TIGRFAMs v15.0 databases [29]. These 122 archaeal marker proteins were selected following previously established criteria [30]. The phylogenetic tree was constructed using IQ-TREE2 [22] (-s example.phy -m MFP -B 1000 --bnni -T AUTO), and bootstrap analysis was applied with 1000 replications.

### 2.6. AlphaFold3 Prediction, Structure Comparison and Docking Analysis

The three-dimensional (3D) structure of the MatE transporter from a previously reported *M. shengliensis* strain was selected as a structural reference [20], and structurally aligned with the experimentally resolved archaeal MATE transporter PfMATE from *Pyrococcus furiosus* (PDB ID: 3VVN). Structural deviation was quantified by calculating the root mean square deviation (RMSD), yielding a value of 0.713 Å. The 3D structures of 853 MatE homologs from representative strains were predicted using AlphaFold3 (https://alphafoldserver.com/, accessed on 15 January 2025). The quality of predicted structures was evaluated using pLDDT scores, which indicated high overall confidence (average score > 90) [31]. All structural visualizations were generated using PyMOL (http://www.pymol.org/, accessed on 24 March 2025). Small-molecule ligands were obtained from the PubChem database (https://pubchem.ncbi.nlm.nih.gov/, accessed on 26 May 2025) and preprocessed using Molecular Operating Environment (MOE) version 2022.02 to generate appropriate conformations and isomers [32]. The processed ligands were saved in the ligand database in “.pdb” format. Receptor protein structures were obtained from AlphaFold (https://alphafoldserver.com/, accessed on 15 January 2025), further refined using the QuickPrep module in MOE, and their active sites were identified using the Site Finder tool [33]. The triangular matcher method was used for ligand placement, with London dG applied during this stage. An Induced Fit model was employed for refinement, utilizing GBVI/WSA dG in this phase [34]. The placement and refinement steps were set to generate 50 and 20 positions, respectively, before initiating the docking process.

### 2.7. Statistical Analysis

All statistical analyses and graphical visualizations were performed using GraphPad Prism 10 (GraphPad Software, San Diego, CA, USA). One-way analysis of variance (ANOVA) with Welch’s correction was applied for all group comparisons to account for unequal variances. Data are presented as mean ± standard deviation unless otherwise stated. A *p* value < 0.05 was considered statistically significant.

## 3. Results and Discussion

### 3.1. Diversity of MatE Transporters in Archaea

**Figure 1:**
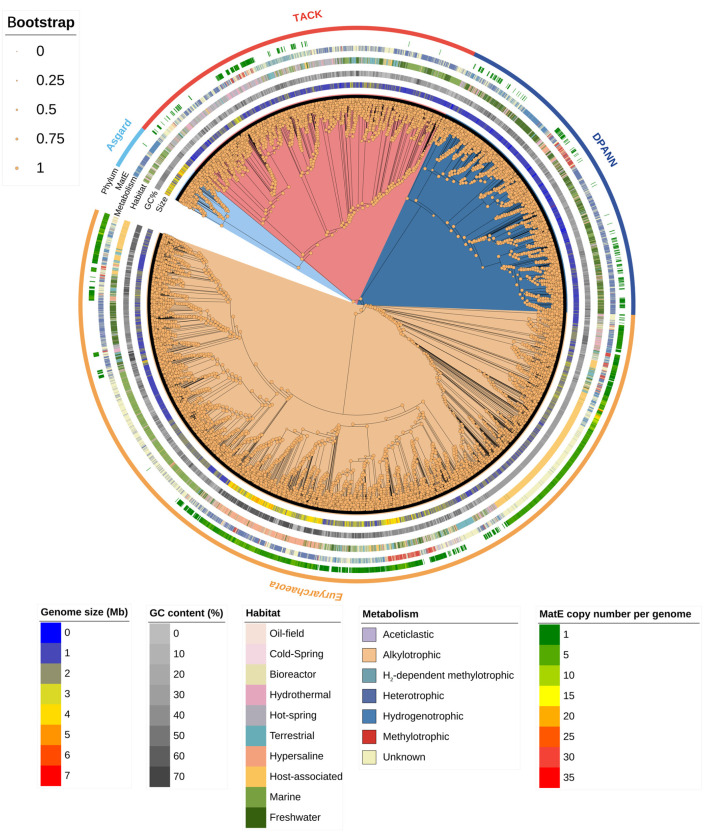
Phylogenetic tree of archaea. A maximum likelihood phylogenetic tree was constructed based on 10,864 archaeal genomes (see Table S1). The outer rings indicate genome size (Mb), GC content (%), habitat categories, metabolic types, and the copy number of MatE-encoding genes per genome, based on a total of 4351 MatE proteins identified across the dataset. Blue, red, navy blue, and orange denote members of the Asgard, TACK, DPANN, and *Euryarchaeota* supergroups, respectively. Alkylotrophic and heterotrophic are used as descriptive categories based on genome annotations or literature evidence, whereas H_2_-dependent methylotrophic, aceticlastic, hydrogenotrophic, and methylotrophic metabolisms represent canonical archaeal metabolic pathways. Genomes with unassigned metabolic features are categorized as Unknown. Bootstrap support values were calculated from 1000 replicate trees and are indicated by node color intensity. Black lines represent phylogenetic branches.

To systematically examine the diversity of MatE transporters in archaea, we performed a large-scale comparative genomic analysis based on a well-annotated archaeal phylogenetic framework comprising 10,864 genomes spanning four major superphyla: Asgard, TACK (*Thaumarchaeota*, *Aigarchaeota*, *Crenarchaeota*, and *Korarchaeota*), DPANN (*Diapherotrites*, *Parvarchaeota*, *Aenigmarchaeota*, *Nanohaloarchaeota*, and *Nanoarchaeota*), and *Euryarchaeota* [2]. Among the analyzed genomes, 4351 archaeal strains encode at least one MatE transporter, whereas 6513 genomes lack detectable MatE homologs (Figure 1 and Supplementary Figure S1). This uneven distribution indicates substantial variability in MatE presence across archaeal lineages. In parallel, we observed notable differences in genome size across environmental categories. For example, archaeal genomes from hypersaline environments tend to be relatively larger (0.37–7.28 Mb, with most around 2.8 Mb), whereas genomes from freshwater-associated archaea are generally smaller (0.29–5.17 Mb, with most around 1.2 Mb). These patterns suggest that genome architecture varies considerably among archaeal populations occupying distinct ecological niches. In addition, we also observed an association between genomic GC content and phylogenetic placement: closely related strains tend to exhibit similar GC contents (Figure 1). Archaeal lineages sharing similar habitats and metabolic features tend to cluster locally within the phylogenetic tree, separated by small numbers of branches, but they are relatively scattered in terms of overall evolutionary relationships. Thus, patterns of ecological distribution, genome architecture, and metabolic potential in archaea cannot be fully explained by phylogenetic relationships alone, suggesting that factors beyond phylogenetic trees contribute to archaeal ecological diversification.

### 3.2. Enriched MatE Transporter in a Specific Habitat

**Figure 2:**
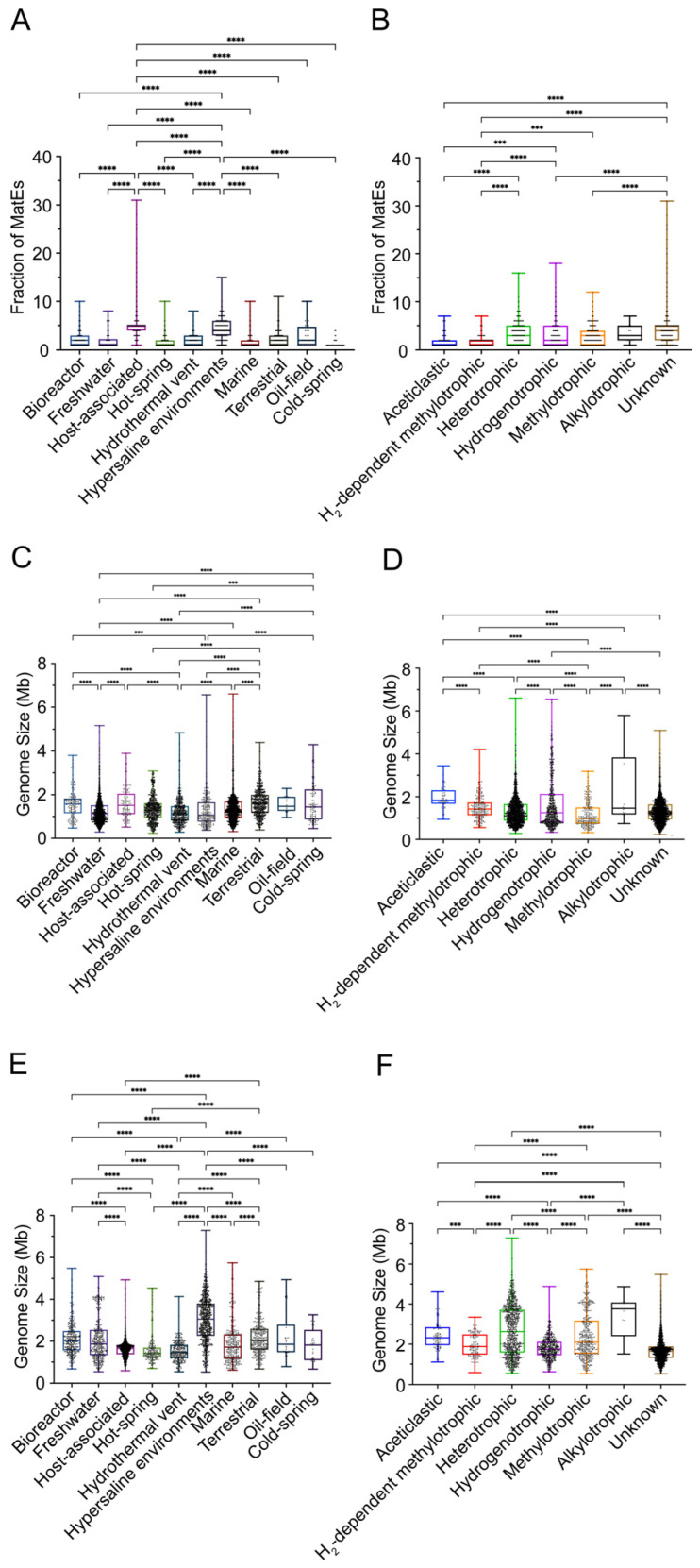
MatE transporters are associated with archaeal habitats and metabolic strategies. (**A**) Abundance of MatE transporters across different habitats among 4351 archaeal genomes containing MatE. (**B**) Abundance of MatE transporters across different archaeal metabolic types among the same 4351 MatE-containing genomes. Alkylotrophic and heterotrophic are used as descriptive categories based on genome annotations or literature evidence, whereas H_2_-dependent methylotrophic, aceticlastic, hydrogenotrophic, and methylotrophic metabolisms represent canonical archaeal metabolic pathways. Genomes with unassigned metabolic features are categorized as Unknown. (**C**) Relationship between habitats and genome size in 6513 archaeal genomes lacking MatE transporters. (**D**) Relationship between metabolic types and genome size in the same 6513 MatE-lacking genomes. (**E**) Relationship between habitats and genome size in 4351 MatE-containing archaeal genomes. (**F**) Relationship between metabolic types and genome size in the same 4351 MatE-containing genomes. Panels (**C**,**D**) serve as control groups (genomes lacking MatE), whereas panels (**E**,**F**) represent experimental groups (genomes containing MatE). Colors indicate different habitat categories or metabolic types as labeled on the x-axis. In each boxplot, the median is shown in the box as a thick bar (unless it coincides with the borderline). The 25th and 75th percentiles are, respectively, in the lower and upper bounds of the box. Lines through the boxes indicate the minimum value and maximum values, while the whiskers correspond to the 1.5 interquartile range from the bounds. Asterisks indicate levels of statistical significance determined by one-way ANOVA with Welch’s correction (***, *p* < 0.001; ****, *p* < 0.0001).

To further investigate the ecological distribution of MatE transporters in archaea, we systematically integrated the niche information and metabolic type annotation for all analyzed genomes. Based on the available metadata, archaeal strains were classified into ten habitat types and six metabolic categories. To control for potential habitat- or metabolism-driven biases, genomes encoding MatE transporters were compared with genomes lacking MatE homologs within the same ecological environment and metabolic categories. Across habitats, MatE transporters exhibited a non-random distribution. They were significantly more abundant in host-associated and hypersaline environments than in other habitats, whereas genomes from oil-field and cold-spring environments showed markedly lower abundance, indicating a clear environmental preference (Figure 2A). A similar pattern was observed across metabolic types: MatE transporters were mainly enriched in heterotrophic, hydrogenotrophic, and methylotrophic archaea, but were substantially less abundant in H_2_-dependent methylotrophic, aceticlastic, and alkylotrophic archaea (Figure 2B). These results suggest that MatE expansion may be linked to specific energy metabolism strategies, particularly those involving diverse organic substrates.

To evaluate whether these patterns could be explained solely by habitat or metabolic composition, we further compared genome size and GC content between MatE-lacking and MatE-containing genomes under identical ecological and metabolic stratifications (Figure 2C–F and Supplementary Figure S2). MatE-lacking genomes were primarily found in marine and freshwater environments and among heterotrophic archaea. These genomes were relatively compact, with median sizes of approximately 1.2 Mb in marine environments, 1.4 Mb in freshwater environments, and 1.2 Mb in heterotrophic archaea, although maximum sizes reached up to 6.6 Mb (Figure 2C,D). Their GC content showed a narrow distribution, clustering around ~40% (Supplementary Figure S2A,B). In contrast, MatE-containing genomes displayed distinct genomic features under the same ecological and metabolic classifications. These genomes were primarily distributed in host-associated and hypersaline environments and among heterotrophic archaea, with substantially larger median genome sizes of approximately 2.6 Mb, 3.7 Mb, and 2.8 Mb, respectively, and maximum sizes approaching 7.5 Mb (Figure 2E,F). GC content also varied across habitats, with median values of approximately 32% in host-associated environments and 65% and 62% in hypersaline environments and heterotrophic archaea, respectively (Supplementary Figure S2C,D). Overall, these results indicate that the distribution of MatE transporters in archaea is non-random and consistently associated with specific habitats and metabolic types, reflecting coordinated patterns with genome size and GC content.

### 3.3. Relationship Between Genome Size and MatE Abundance

**Figure 3:**
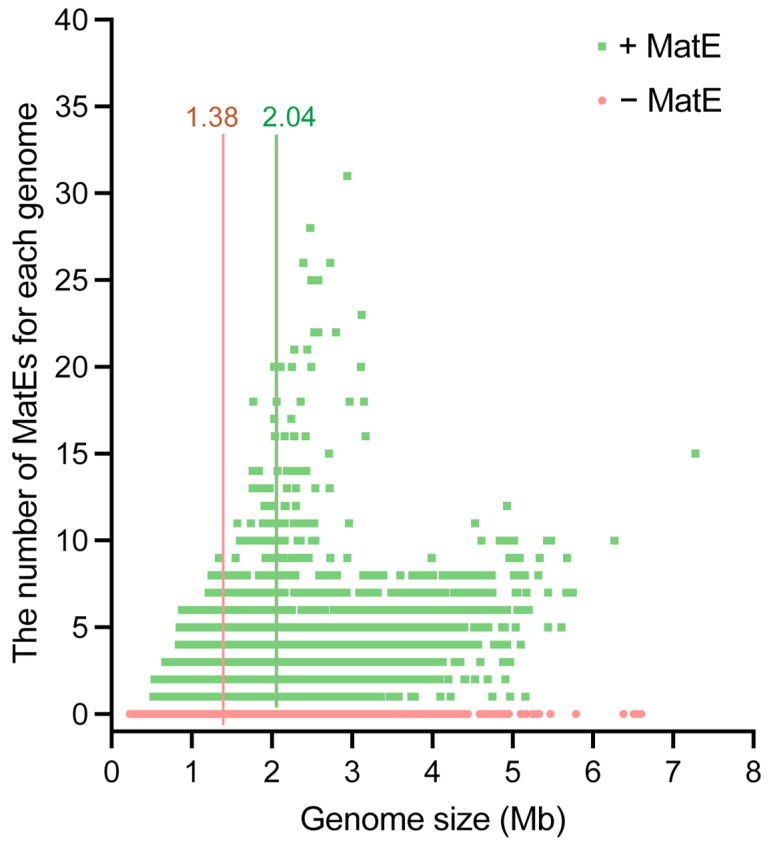
Correlation between MatE number and genome size. Relationship between the number of MatEs and the genome size, which varies from 0.23 to 7.28 Mb. The green dots represent strains with MatEs, and the green line represents their median number; the red dots represent strains that lack MatEs, and the red line represents their median number. “+ MatE” and “– MatE” indicate strains that do and do not contain MatE, respectively. Each point represents a single archaeal genome. Genomes lacking MatE transporters are shown at zero abundance.

To assess whether the uneven distribution of MatE transporters in archaeal genomes can be attributed to general genome traits, we further analyzed the relationship between genome size and the abundance of MatE transporters. The results showed that genomes lacking MatE were distributed over the entire genome size range, and their MatE abundance was 0 (Figure 3). The average genome size of the strains containing MatE was 2.04 Mb, which was significantly higher than that of the strains without MatE (Figure 3). The results indicate that the presence of MatE transporters is related to the genome size of archaeal, and their uneven distribution is not random but tends to occur in strains with larger genomes.

### 3.4. Variations in Habitats Account for the Structural Diversity of MatE

**Figure 4:**
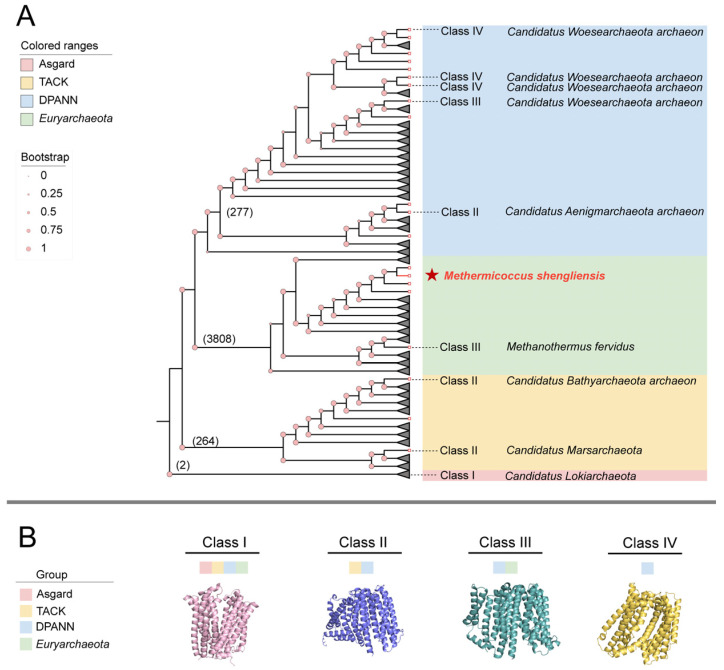
Diversity and distribution of MatE transporters. (**A**) Phylogenetic tree of MatE proteins. The numbers in parentheses indicate the number of archaeal MatE proteins identified in each superphylum (Asgard, TACK, DPANN and *Euryarchaeota*). Based on structural configurations, MatE transporters are classified into four types (Class I to Class IV), with unannotated types considered as Class I. The phylogenetic tree was constructed using the maximum likelihood method with 1000 bootstrap replicates. Tree visualization was performed using iTOL (https://itol.embl.de/, accessed on 12 June 2025). (**B**) Overall struures of different MatE proteins.

To study the evolutionary distribution and structural diversity of archaeal MatE transporters, we used phylogenetic analysis and in combination with protein structure prediction using AlphaFold3 (https://alphafoldserver.com/, accessed on 15 January 2025) [35]. Archaea encong MatE transporters s were divided into four superphyla: Asgard (2 strains), TACK (264 strains), DPANN (277 strains), and *Euryarchaeota* (3808 strains) (Figure 4A). A total of 853 representative MatE proteins were selected from the above groups (Table S3) and compared with the structure of MatE from *M. shengliensis.* Based on structural clustering analysis, archaeal MatE transporters were classified into four distinct structural classes, designated Class I, Class II, Class III and Class IV (Figure 4B). Phylogenetic analysis showed that Class I MatE transporters are mainly distributed in TACK, *Euryarchaeota*, and DPANN superphyla, occupying a major proportion in different habitats and metabolic types. In contrast, Classes II, III, and IV members were distributed in multiple phylogenetic branches, but were less abundant in all habitats and metabolic types (Figure 4 and Supplementary Figure S3). Moreover, functional prediction annotations show that the Class I MatE transporters are primarily annotated as MATE family efflux transporter, whereas Class II, Class III, and Class IV are annotated as oligosaccharide flippase family protein, polysaccharide biosynthesis protein, and flippase, respectively (Table S3). Taken together, these results show that archaeal MatE transporters are dominated by a single, widely distributed structural class (Class I), which accounts for approximately 99% of the analyzed proteins, whereas Classes II–IV are rare and sporadically distributed.

### 3.5. MatE Transporters Recognize and Interact with Diverse Substrates Through Specific Amino Acid Residues

**Figure 5:**
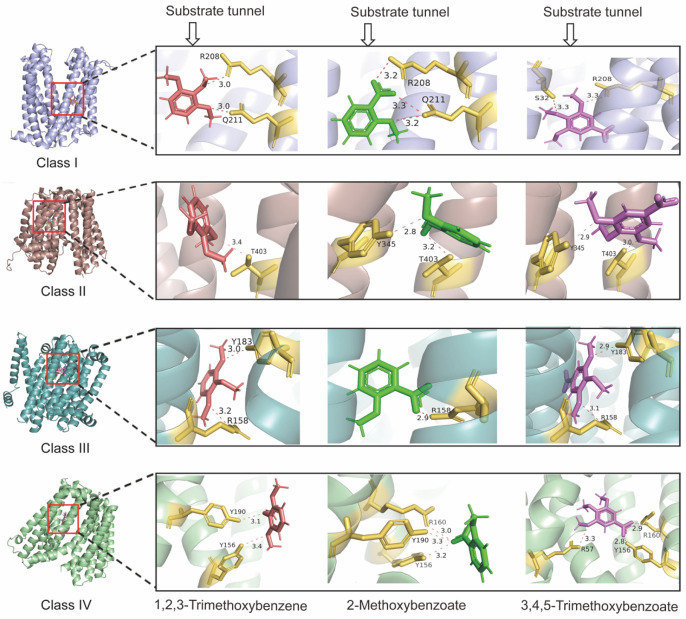
Molecular docking of MatE transporters with different substrates. The figure shows the key residues at the active sites of Class I, Class II, Class III, and Class IV MatE structures when interacting with the substrates 1,2,3-Trimethoxybenzene, 2-Methoxybenzoate, and 3,5-Trimethoxybenzoate. Protein backbones are shown in cartoon representation and colored differently for each class. Substrates are shown as stick models and colored red (1,2,3-trimethoxybenzene), green (2-methoxybenzoate),and purple (3,4,5-trimethoxybenzoate). Key interacting residues are shown as yellow sticks. Red dashed lines indicate hydrogen bonds, , and the numbers next to the dashed lines represent the distances between atoms or residues in the protein, measured in Ångströms (Å).

In order to analyze the interaction between different structural classes of archaeal MatE transporters and various substrates, molecular docking analyses were performed. Representative structures of MatE Classes I–IV were docked with a variety of potential substrates [36,37,38], including methanol, methylamine, dimethyl sulfide, 1,2,3-trimethoxybenzene, 2-methoxybenzoate, and 3,4,5-trimethoxybenzoate (Figure 5 and Table S4). For substrate molecules such as methanol, methylamine, and dimethyl sulfide, stable docking conformations were not obtained, which may be related to their small molecular weight and the difficulty of forming stable interactions in the predicted binding cavity. In contrast, the larger methoxylated aromatic compounds exhibited a recognizable binding mode in all four classes of MatE (Figure 5). Notably, there is a specific residue in each class of MatE structure that consistently participates in the interactions with three methoxylated aromatic compounds: R^208^ in Class I, T^403^ in Class II, R^158^ in Class III, and Y^156^ in Class IV (Figure 5). To further evaluate whether other types of substrates exhibit similar interaction patterns with the MatE transporters, antibiotic norfloxacin and polysaccharide raffinose were selected for molecular docking analysis with four classes of MatE structures, respectively (Supplementary Figure S4). In Class I transporters, residues W^258^ and K^435^ were found to interact with both norfloxacin and raffinose. In Classes II and III, the residues T^403^ and R^158^, previously involved in binding methoxylated aromatic compounds, were also involved in raffinose docking (Figure 5 and Supplementary Figure S4). However, in Class IV, the residues interacting with norfloxacin (R^145^) and raffinose (F^50^ and E^63^) did not overlap with those involved in methoxylated aromatic compound binding (Supplementary Figure S4). Taken together, these molecular docking results indicate that archaeal MatE transporters with different structural classes have significant differences in the predicted interaction patterns of substrates and residues.

## 4. Conclusions

In this study, we performed a large-scale comparative genomic and structural analysis of MatE transporters across 10,864 archaeal genomes spanning four major superphyla. MatE homologs were detected in 4351 genomes, with a markedly uneven, non-random distribution across phylogenetic lineages, habitats, and metabolic types. Comparative analyses between of MatE-positive and MatE-negative genomes revealed consistent enrichment of MatE in host-associated and hypersaline environments, as well as associations with heterotrophic, hydrogenotrophic, and methylotrophic lifestyles. Notably, MatE-positive genomes displayed larger genome sizes, suggesting that MatE occurrence is more closely related to extended genome structure than to habitat alone.

Structural analyses showed that archaeal MatE transporters are dominated by a single structural class (Class I; ∼99% of sequences), while Classes II–IV are rare and sporadically distributed across phylogeny, habitats, and metabolisms, underscoring strong evolutionary conservation of Class I. Molecular docking further revealed class-specific substrate–residue interaction patterns, with distinct key residues implicated in ligand binding, suggesting functional differentiation among MatE Classes.

Collectively, these findings link the distribution, structural conservation, and predicted substrate interactions of MatE transporters to ecological niches, metabolic strategies, and genome size, highlighting the role of ecological constraints in shaping archaeal evolutionary patterns and habitat adaptation.

## Data Availability

The original contributions presented in this study are included in the article/Supplementary Material. Further inquiries can be directed to the corresponding author.

## Supplementary Materials

The following supporting information can be downloaded at https://www.mdpi.com/article/10.3390/microorganisms14030531/s1, Figure S1. Phylogenetic tree of MatE proteins from 4351 archaeal genomes. Figure S2. GC content variation across habitats and metabolic types in archaeal genomes with and without MatE. Figure S3. MatEs are involved in habitat adaptation. Figure S4. Molecular docking of different classes of MatE transporters with norfloxacin and raffinose. Table S1. Genomes used to construct the archaea reference tree. Table S2. Phylogenetic tree of MatE proteins. Table S3. Results of structural clustering of MatE proteins. Table S4. Information on representative MatE proteins from different structural clusters. https://doi.org/10.5281/zenodo.18079802 (accessed on 29 December 2025) [36,37,38].
