# Supplementary material for: Comparative Genomic Insights into MatE Transporter Diversity and Habitat Adaptation of Archaea
Source: Microorganisms. 2026 Feb 25;14(3):531. doi: 10.3390/microorganisms14030531 (PMC13028788; doi:10.3390/microorganisms14030531)
Supplement: Supplementary file 1 [file microorganisms-14-00531-s001.zip › Supplementary Figure.pdf]

Supplementary Figures

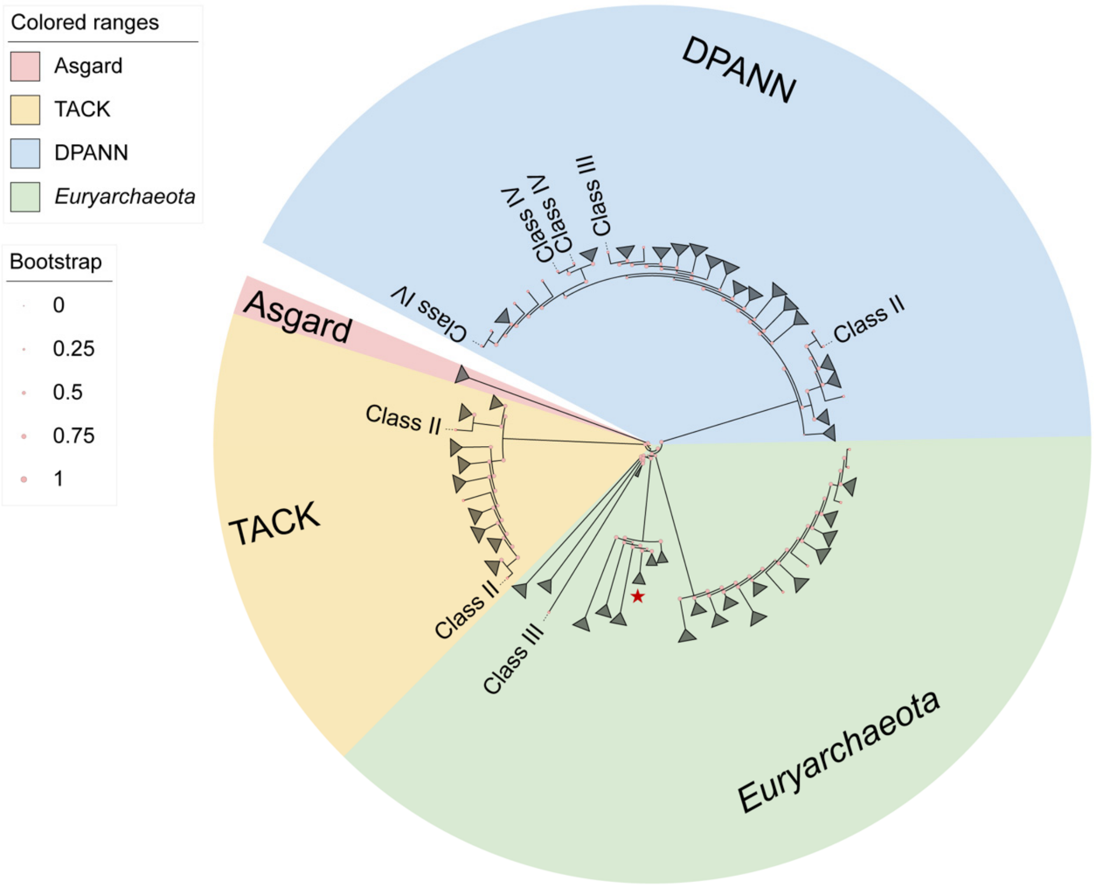

Figure S1. Phylogenetic tree of MatE proteins from 4,351 archaeal genomes. The phylogenetic tree was constructed using all MatE proteins obtained from 4,351 MatE-containing archaeal genomes. For structural classification, a subset of 853 representative MatE proteins was selected, and these strains are classified into four structural classes (Class I–IV). Strains not labeled on the tree are assigned to Class I. Red, yellow, blue, and green indicate the subgroups of Asgard, TACK, DPANN, and *Euryarchaeota*, respectively. The reference *Methermicoccus shengliensis* MatE is indicated by a red star.

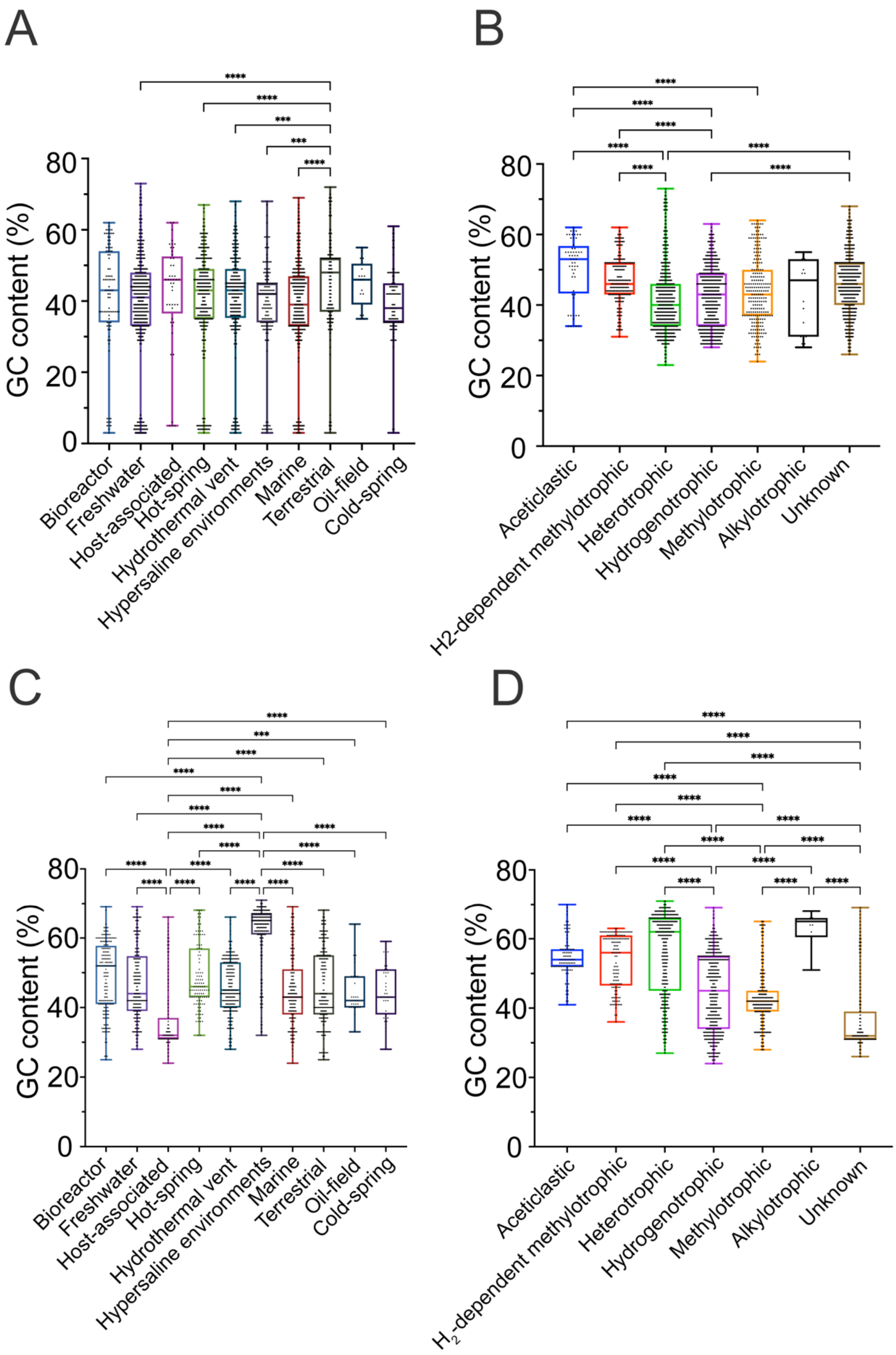

Figure S2. GC content variation across habitats and metabolic types in archaeal genomes with and without MatE. (**A**) GC content distributions across habitats in 6,513 archaeal genomes lacking MatE transporters (controls). (**B**) GC content

distributions across archaeal metabolic types in the same 6,513 MatE-lacking genomes. Alkylotrophic and heterotrophic are used as descriptive categories based on genome annotations or literature evidence, whereas H_2-dependent methylotrophic, aceticlastic, hydrogenotrophic, and methylotrophic metabolisms represent canonical archaeal metabolic pathways. Genomes with unassigned metabolic features are classified as Unknown. (**C**) GC content distributions across habitats in 4,351 archaeal genomes containing MatE transporters. (**D**) GC content distributions across metabolic types in the same 4,351 MatE-containing genomes. Panels **A** and **B** serve as control groups, whereas panels **C** and **D** represent experimental groups. In each boxplot, the median is shown in the box as a thick bar (unless it coincides with the borderline). The 25th and 75th percentiles are, respectively, in the lower and upper bounds of the box. Lines through the boxes indicate the minimum value and maximum values, while the whiskers correspond to the 1.5 interquartile range from the bounds. Asterisks indicate levels of statistical significance determined by one-way ANOVA with Welch's correction (*, $P < 0.05$; **, $P < 0.01$; ***, $P < 0.001$; ****, $P < 0.0001$; ns represent no significant difference).

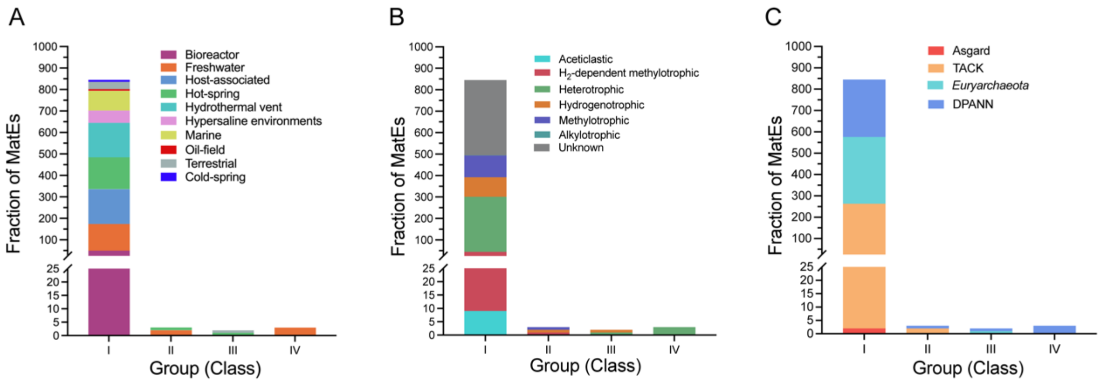

Figure S3. MatEs are involved in habitat adaptation. (**A**) The distribution of the four MatE structural classes (Class I–Class IV) across ten different ecological niches in the bar chart. (**B**) The distribution of the four MatE structural classes across six metabolic types in the bar chart. (**C**) The distribution of the four MatE structural classes (Class I–Class IV) across the four archaeal superphyla in the bar chart.

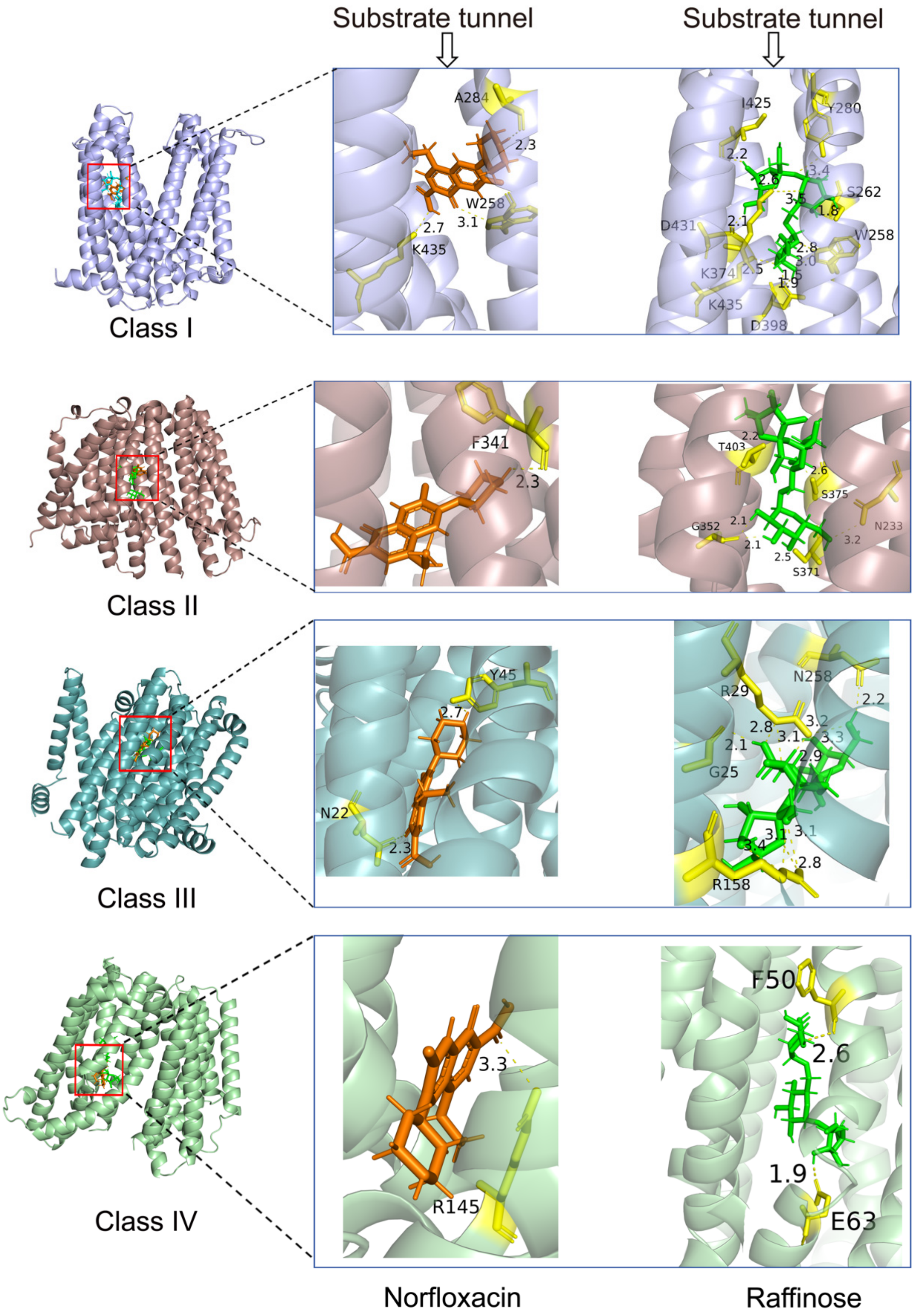

Figure S4. Molecular docking of different classes of MatE transporters with norfloxacin and raffinose. Yellow dashed lines indicate hydrogen bonds, and the numbers adjacent to the dashed lines represent the distances between interacting atoms or residues in the protein, measured in ångströms (Å).
